# Use of behavioral economics and social psychology to improve treatment of acute respiratory infections (BEARI): rationale and design of a cluster randomized controlled trial [1RC4AG039115-01] - study protocol and baseline practice and provider characteristics

**DOI:** 10.1186/1471-2334-13-290

**Published:** 2013-06-27

**Authors:** Stephen D Persell, Mark W Friedberg, Daniella Meeker, Jeffrey A Linder, Craig R Fox, Noah J Goldstein, Parth D Shah, Tara K Knight, Jason N Doctor

**Affiliations:** 1 Division of General Internal Medicine and Geriatrics, Institute for Healthcare Studies, Feinberg School of Medicine, Northwestern University, 750 N. Lake Shore Drive, 10th Floor, 60611, Chicago, IL, USA; 2 RAND, 20 Park Plaza, Suite 920, Boston, MA 02116, USA; 3 RAND, 1776 Main St., Santa Monica, CA 9040, USA; 4 Division of General Medicine and Primary Care, Brigham and Women’s Hospital; Harvard Medical School, Boston, MA 02116, USA; 5 Department of Psychology, UCLA Anderson School of Management, David Geffen School of Medicine at UCLA, 110 Westwood Plaza D-511, Los Angeles, CA 90095, USA; 6 Department of Health Behavior, UNC Gillings School of Global Public Health, 308 Rosenau Hall, Campus Box 7440, Chapel Hill, NC 27599-7440, USA; 7 Leonard D. Schaeffer Center for Health Policy and Economics, University of Southern California, 3335 S. Figueroa Street, Unit A, Los Angeles, CA 90089-7273, USA

**Keywords:** Antibiotics, Acute respiratory infections, Behavioral economics, Social psychology, Clinical decision support

## Abstract

**Background:**

Inappropriate antibiotic prescribing for nonbacterial infections leads to increases in the costs of care, antibiotic resistance among bacteria, and adverse drug events. Acute respiratory infections (ARIs) are the most common reason for inappropriate antibiotic use. Most prior efforts to decrease inappropriate antibiotic prescribing for ARIs (e.g., educational or informational interventions) have relied on the implicit assumption that clinicians inappropriately prescribe antibiotics because they are unaware of guideline recommendations for ARIs. If lack of guideline awareness is not the reason for inappropriate prescribing, educational interventions may have limited impact on prescribing rates. Instead, interventions that apply social psychological and behavioral economic principles may be more effective in deterring inappropriate antibiotic prescribing for ARIs by well-informed clinicians.

**Methods/design:**

The Application of Behavioral Economics to Improve the Treatment of Acute Respiratory Infections (BEARI) Trial is a multisite, cluster-randomized controlled trial with practice as the unit of randomization. The primary aim is to test the ability of three interventions based on behavioral economic principles to reduce the rate of inappropriate antibiotic prescribing for ARIs. We randomized practices in a 2 × 2 × 2 factorial design to receive up to three interventions for non-antibiotic-appropriate diagnoses: 1) Accountable Justifications: When prescribing an antibiotic for an ARI, clinicians are prompted to record an explicit justification that appears in the patient electronic health record; 2) Suggested Alternatives: Through computerized clinical decision support, clinicians prescribing an antibiotic for an ARI receive a list of non-antibiotic treatment choices (including prescription options) prior to completing the antibiotic prescription; and 3) Peer Comparison: Each provider’s rate of inappropriate antibiotic prescribing relative to top-performing peers is reported back to the provider periodically by email. We enrolled 269 clinicians (practicing attending physicians or advanced practice nurses) in 49 participating clinic sites and collected baseline data. The primary outcome is the antibiotic prescribing rate for office visits with non-antibiotic-appropriate ARI diagnoses. Secondary outcomes will examine antibiotic prescribing more broadly. The 18-month intervention period will be followed by a one year follow-up period to measure persistence of effects after interventions cease.

**Discussion:**

The ongoing BEARI Trial will evaluate the effectiveness of behavioral economic strategies in reducing inappropriate prescribing of antibiotics.

**Trials registration:**

ClinicalTrials.gov: NCT01454947

## Background

Acute respiratory infections (ARIs) constitute about 10% of all ambulatory care visits in the United States and account for 44% all antibiotic prescriptions provided in ambulatory care [1]. Despite the fact that the vast majority of ARIs in adults are caused by viruses, antibiotic use for ARIs remains common [1,2]. Although the Centers for Disease Control and Prevention and others have placed increased emphasis on reducing inappropriate antibiotic use, prescribing rates declined only modestly between 1995 and 2006, and the use of broader-spectrum antibiotics increased [1].

Clinicians who prescribe antibiotics for non-bacterial infections expose patients to unnecessary risks of adverse drug events, and increased costs [3]. Furthermore, antibiotic overuse increases the spread of antibiotic-resistant bacteria which have become a major public health problem [2,4,5]. Interventions to reduce antimicrobial prescribing such as physician and patient education, physician audit and feedback, and financial or regulatory incentives have only been modestly successful, generally producing 10% reductions in antibiotic prescribing rates [6,7]. Educational interventions may have limited impact on prescribing rates if lack of guideline awareness is not the primary reason for inappropriate antibiotic prescribing.

Recognizing the limitations of educational and informational interventions, we developed novel interventions, drawing on insights from behavioral economics and social psychology, designed to appeal to provider self-image and social motivation and thereby produce larger and more enduring effects. These interventions take into account a growing body of research indicating that individuals act within broad social contexts and behave in ways that are not always rational but may be predictable [8-11].

This article discusses the rationale, design, development, and implementation of these interventions as part of a cluster randomized controlled trial to evaluate the use of behavioral economics in the treatment of ARIs.

## Methods/design

### Study aims

The main intent of this study is to determine whether interventions that leverage information technology and apply behavioral economic concepts reduce the rate of antibiotic prescribing for ARIs. Our primary hypothesis is that practices randomized to receive behavioral economic interventions will have lower antibiotic prescribing rates for non-antibiotic appropriate ARIs compared to control practices. We further hypothesize that for the treatment conditions, individual prescribers’ rates of antibiotic prescribing for encounters with a non-antibiotic appropriate ARI diagnoses will decrease relative to their own historical control rates.

### Practice settings, physician recruitment and consent

We recruited physicians and advance practice nurses from 49 primary care clinics affiliated with three healthcare organizations. Providers were eligible if they treated adult patients with acute respiratory infections. Each study clinic was required to have an electronic health record (EHR) system in place and had to have its own physical building (as opposed to multiple clinics sharing the same space, such as the floor of a hospital, where interactions between providers assigned to different intervention groups would be more likely).

With the assistance of each site’s medical director, we sent providers at participating sites an introductory email that included a description of the broad goals of the study, a general description of the intervention, and a link to the electronic consent form and baseline survey. We also described compensation providers would receive for participation. The leadership of each clinic site decided whether the financial incentives to participate would be paid as a stipend to the clinic as a whole or to the individual clinician. The amount of the compensation provided to the clinic per participating provider was approximately the same regardless of whether the clinic was randomized to no intervention, one intervention, or multiple interventions ($1200 per full-time provider). We sent up to 6 (median of 3) follow up emails to providers who did not respond, and study personnel contacted them in person when feasible.

Participating providers completed written informed consent. The consent document indicated that participation was voluntary and that decisions to participate (or not) would have no bearing on any provider’s status at his or her clinic. Providers who gave consent to participate were asked to complete an online survey and brief educational session prior to the intervention phase, permit de-identified patient records pertaining to patients who saw them for ARIs to be included in the study database, and complete a 15 minute post-intervention survey (See Additional file 1: Appendices A and B).

The study protocol for all clinic sites was reviewed and approved by the University of Southern California’s Institutional Review Board (IRB). Individual protocols for Massachusetts and California sites were reviewed and approved by their local IRBs.

### Provider education & baseline survey

After providing consent, all providers were asked to complete a 15 to 20 minute online survey and educational module. The educational module contained information about ARIs derived from evidence based guidelines and systematic reviews [12-21]. The educational module also described the interventions to which a clinician’s site was assigned, including changes they would observe in their EHR (for Accountable Justifications and Suggested Alternatives interventions) and examples of the kinds of emails they would receive (Peer Comparison). Additional file 1: Appendix A provides examples of the content of these enrollment and education materials.

### Interventions

We developed intervention components (including clinical decision support rules, planned workflows, and computerized order sets) using current guidelines for ARIs, as well as input from a research team comprised of individuals with expertise in primary care, acute respiratory infections, behavioral economics, clinical informatics, and the capabilities of the existing EHR systems used by each study site. The clinical decision support was customized according to the capabilities of the three EHR systems in use (NextGen, NextGen Healthcare, Horsham, PA; EpicCare, Epic Systems Co., Verona, WI; and the Longitudinal Medical Record, Partners Healthcare, Boston, MA). All interventions were designed to minimize disruption in provider workflow and to avoid limiting the treatment options available.

### EHR-based interventions

**Figure 1:**
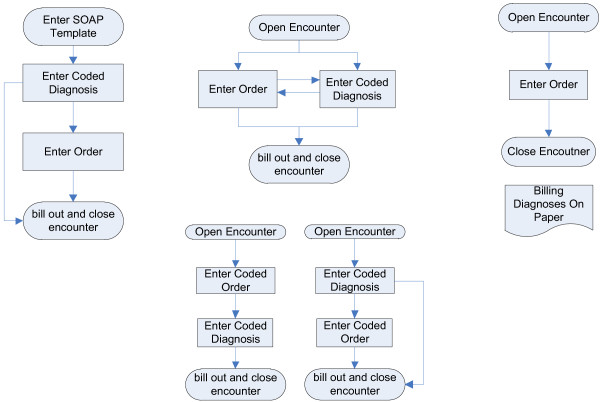
Work flow schema for the 3 electronic health records used.

The research team developed model workflows described below for the two clinical decision support interventions (*Accountable Justifications* and *Suggested Alternatives*). These model workflows were iteratively modified to accommodate technical constraints and organizational preferences for each of the three organizations. The Figure 1 illustrates the three workflows relevant to the design of the study for each of the three EHRs used. Further accommodations were required to support the group randomized in the factorial design to receive both interventions. Additional file 1: Appendix C contains the details of development and customization that was required at each site.

### Accountable justifications intervention

#### Rationale for accountable justifications

In the Accountable Justifications intervention, clinicians are prompted to record an explicit justification for why they are prescribing an antibiotic to a patient with an ARI that appears in the patient’s EHR. Accountable justifications incorporate several behavioral principles. First, they signal an injunctive norm (a norm, often provided by an authoritative source, that strongly indicates how people should behave) indicating that prescribing an antibiotic is not recommended. This may make the provider more likely to believe both that not prescribing an antibiotic is the best medical decision and that prescribing when it is not indicated violates professional standards. Second, they incorporate social accountability. Provider justifications become an explicit, separate part of the medical record, so a provider’s decision to prescribe is subject to the review and judgment of the provider’s peers [22-24]. Third, the justification alert implicitly designates guideline-concordant prescribing as the default action. Defaults are options that are exercised if the decision maker takes no special action to opt in or out of a given choice [25-28]. Prior to our intervention, choosing to deviate from guidelines did not carry a special requirement to document a clinical rationale in the EHR. Accountable justifications, therefore, reset the default action. Guideline-concordant treatment choices (i.e., not prescribing an antibiotic for an ARI) continue to require no special justification, but a provider must now “opt-in” to prescribing an antibiotic by providing a justification for which they are accountable.

Defaults may affect behavior for a number of reasons: 1) they may be perceived as a recommended action; 2) they require less effort (in this case, by following the default a provider can avoid the workflow disruption caused by the justification alert); 3) they serve as a reference point so that relative disadvantages of alternatives are viewed as losses that loom larger than relative advantages; and 4) they may seem less anxiety-inducing as people tend to regret active choices that lead to poor outcomes more than they regret failures to act that lead to poor outcomes. We expect all of these factors to contribute to a shift away from inappropriate antibiotic prescribing [25,26].

#### Model workflow for delivery of accountable justifications

When a provider orders an antibiotic through the EHR and enters a non-antibiotic appropriate ARI diagnosis (listed in Table 1) a computerized alert appears (Additional file 1: Appendix D). The alert briefly summarizes the guideline corresponding to the ARI diagnosis for which the antibiotic is being written (e.g., antibiotics are not indicated for non-specific upper respiratory infections). If the provider persists in wanting to prescribe an antibiotic, the alert asks the provider to check a box stating “If you still want to prescribe an antibiotic, please check the box and write your reason for doing so.” The clinician must write his or her justification for prescribing the antibiotic in a free-text field in the EHR; no pre-written justifications are given. The alert informs the provider that the free-text justification he or she provides will be inserted into the patient’s medical record where other providers can see it, and that if he or she does not enter a justification, the statement “No justification for prescribing antibiotics was given” will be entered into the patients’ medical record. If the antibiotic order is canceled no justification is required, and this text will not appear.

**Table 1:** Acute Respiratory Infection (ARI) diagnoses related to interventions and outcomes assessments

**Diagnoses**		**ICD9-CM**	**Used to trigger decision support**
**Non-antibiotic appropriate ARI diagnoses (Included in Peer Comparison and Primary Outcome Assessment)**			
	Acute nasopharyngitis (common cold)	460	Non-specific URI
	Acute laryngitis and tracheitis	464, 464.0, 464.00, 464.1, 464.10, 464.2, 464.20, 464.4, 464.50	Non-specific URI
	Acute laryngeopharyngitis/acute upper respiratory infection	465, 465.0, 465.8, 465.9	Non-specific URI
	Acute bronchitis	466, 466.0, 466.1, 466.11, 466.19	Acute bronchitis
	Bronchitis not specified as acute or chronic	490	Acute bronchitis
	Influenza	487, 487.1, 487.8	Influenza
**Potentially antibiotic appropriate ARI Diagnoses (Included in Secondary Outcome Assessment)**			
	Acute sinusitis	461.xx	Acute sinusitis/rhinosinusitis
	Acute pharyngitis	462	Acute pharyngitis
Other ARIs diagnoses or symptoms of interest (Included in Secondary Outcome Assessment)*			
	Streptococcal sore throat	034.0	Acute pharyngitis
	Cough	786.2	Acute bronchitis

* Only additional diagnoses triggering clinical decision support are included here. Additional diagnoses included in the secondary outcomes are listed in Additional file 2: Appendix E.

These alerts are suppressed (i.e., do not appear) when a patient has certain comorbid conditions recorded in the EHR problem list or a past medical history (See Additional file 2: Appendix E). These suppressor diagnoses were compiled by study physicians based on comorbid conditions that may make the clinical guidelines on which the alerts were based less likely to apply to an individual patient.

### Suggested alternatives intervention

#### Rationale for suggested alternatives

When clinicians assigned to the Suggested Alternatives intervention see a patient with an ARI, they receive a list of non-antibiotic treatment choices prior to the time when they would complete an antibiotic prescription. Suggested Alternatives may be effective because one central reason why physicians prescribe antibiotics for ARIs when they are not indicated is perceived pressure from patients requesting a prescription. Patients may be unsatisfied if they do not receive an antibiotic prescription, or at least a prescription for medication of some kind [29]. By making prescription and over-the-counter medications that are alternatives to antibiotics more salient to providers, we facilitate a means by which they can satisfy patient demand for treatment from their provider while at the same time reducing their tendency to prescribe unnecessary antibiotics [26,30].

#### Model workflow for delivery of suggested alternatives

When a provider enters an ARI diagnosis for a patient visit, a computerized alert presents an order set containing multiple non-antibiotic prescription and non-prescription medication choices as well as educational materials that can be printed and given to the patient. These order sets were designed to include many of the most common treatments used to treat ARI symptoms. Additional file 1: Appendix F provides sample content of an order set.

### Peer comparison intervention

#### Rationale for peer comparisons

Social norms are standards that are understood by members of a group and that guide relevant social behavior due to a desire to conform with actual behavior (the descriptive norm) or sanctioned behavior (the injunctive norm) [31]. Numerous studies have shown that people tend to conform to the behavior of others, especially those who are perceived to be similar to one’s self [32]. Such effects have been found in studies of behaviors as diverse as voting, [33] littering, [31] and towel recycling in hotels [34]. Social norms may convey information concerning appropriate behavior or social consequences of failing to conform. Behavioral studies find that these effects persist even when behavior is unobservable (e.g., littering when nobody is around) and when the social information is not particularly informative to one’s own preferences (e.g., towel recycling). We expect that periodically reminding health care providers of their own prescribing behavior, while providing both a descriptive social norm (displaying the behavior of the best performing peers in their region) and an injunctive norm (citing the national recommended guidelines) will lead providers to conform more closely to these norms.

While benchmarks and performance measurement and public reporting have become increasingly common in health care, [35,36] these reports typically include the performance of all providers across the full range of performance. However, providing individuals with statistics of central tendency, such as the statistical mean, can sometimes be ineffective or even backfire for individuals who are currently performing better than the average [37,38]. A seminal study by Kiefe et al. demonstrated that providers who were shown their own performance in relation to 90th percentile performance on measures of preventive and chronic disease care had greater performance improvements than those who were shown their own performance in relation to mean performance [37]. Thus, in the current study those in the Peer Comparison conditions are provided personalized feedback along with the antibiotic over-prescribing rate of only the top performers within their clinic. In addition, injunctive norms (i.e., indicators of socially desirable performance for high performers) are often excluded. These factors suggest that the use of benchmarks can be improved by applying “nudging” interventions with foundations in social decision making.

Our performance feedback reports for each provider randomized to receive the Peer Comparison intervention will have three key characteristics: (1) each target provider will receive his or her individual performance, (2) benchmarks will prominently feature the performance of providers who would be considered credibly peers of the target provider, and (3) benchmarks will reflect *only* performance that is desirable (e.g., showing only the performance of the best-performing credible peers). Prior studies of provider feedback related to antibiotic use for ARIs have not constructed feedback in this way [39-42].

#### Delivery of peer comparisons

In the Peer Comparison intervention, each provider’s individual performance is calculated as the percentage of encounters for non-antibiotic appropriate ARIs listed in Table 1 for which the provider prescribed an oral antibiotic. Encounters occurring with patients who had certain comorbidities or other diagnosed bacterial infections (Additional file 2: Appendix E) are excluded from the calculation. If the provider had more than 20 qualifying ARI encounters in the past 30 days, all these encounters were included in the calculation. Otherwise, the most recent 20 qualifying ARI encounters were included if they occurred in the past 5 months. If fewer than 20 occurred in the past 5 months, only encounters in the past 5 months are included and the provider is excluded from percentile rank calculations.

Providers randomized to Peer Comparison receive, via email, feedback reports presenting their individual performance, updated every 1–2 months. These emails classify their performance as “top performing” (among the 10% of their peers with the “best”—i.e. lowest— prescribing rates) or “not top performing” (for all providers not in the best 10%). The top 10% of participating providers are calculated within each study region: Massachusetts or California. Emails to providers within the top 10% (lowest prescribers) carry a brief message in the subject line: “You are a top performer.” Emails to those not in the top 10% have an equally direct subject line: “You are not a top performer. You are prescribing too many unnecessary antibiotics.” The proportion of “Top Performers” can be greater than 10% of clinicians if more than10% of them had an inappropriate antibiotic prescribing rate of zero.

In addition to this overall classification of performance, the feedback reports for each provider include a denominator count (the number of patient encounters for non-antibiotic appropriate ARIs, after exclusions), a numerator count (the number of denominator patient encounters in which the provider prescribed an oral antibiotic), the provider’s own performance rate (the numerator divided by the denominator), and the performance rate cutoff for the best 10% of providers. Emails also include a link to the guidelines for antibiotic usage in ARIs. Additional file 1: Appendix G provides an example of emails sent to top performers and non-top performers.

### Randomization of study sites

We chose a cluster-randomized design at the clinic level to avoid contamination that might occur if individual providers in close proximity were randomized to different interventions. Providers who practice at multiple clinics were assigned to the intervention of the clinic for which they spend at least 85% of their time. One provider who practiced at multiple clinics was assigned to his own cluster.

Geographically distinct individual clinics were the unit of randomization. Clinics belonged to one of three larger clinical organizations covering a connected geographic area in either Massachusetts (Partners Healthcare consisting of Brigham and Women’s Hospital and Massachusetts General Hospital affiliated primary care practices) or Southern California (AltaMed; The Children’s Clinic—which, despite its name, sees a high volume of adult patients). We carried out a block randomization of clinics by clinic organization using the statistical computing language R [43]. We first constructed two matrices that each represented a main effects design and together represented the full factorial design (2 × 2 × 2 design). For each clinic organization, we constructed ordered collections of clinics. We then employed the sample function in R to return a random permutation of each ordered collection. For each collection of clinic organizations we drew a sample that represented the largest number of clinics within each clinic organization that was divisible by 8, the number of study arms. We then used the list function, a function that ties together related data that do not share the same structure, to assign each randomly permutated clinic to a study arm, repeating this process until clinics had filled the eight arms of the study in equal measure. Because the number of clinics at each organization was not always divisible by eight, there were a total of four “remainder” clinics across all organizations. These remainder clinics were randomized to four conditions within one of the fractional factorial main effects design (a subset of the larger 2 × 2 × 2 design) to maximize power for main effects estimates. This was accomplished in a procedure similar to the one described above. One of the two possible fractional factorial designs of the larger 2 × 2 × 2 design was chosen randomly to assign remainder clinics to a condition so that remainder clinics had an ex ante equal probability of assignment to any one of the eight conditions in the full factorial design. Allocation of the sequence was concealed until after the interventions were assigned.

### Intervention implementation

Dates for intervention implementation differ across geographical location, and were contingent upon variations in organizational structure, provider recruitment procedures, and EHR-specific development times for intervention features. Interventions began at the first sites in November 2011. The interventions will be in place for 18 months at each site. A one year follow up period after the cessation of the interventions is planned to measure persistence of the effects.

### Measurements

We measured provider characteristics and attitudes by survey. We also asked questions about providers’ attitudes toward practice guidelines, clinical decision support, electronic health records, and practice environment. Additional file 1: A provides the content of the provider survey.

### Outcomes

#### Primary outcome

The primary study outcome is whether a provider prescribes an oral antibiotic (excluding antibiotics that are not used to treat any bacterial respiratory pathogens, Additional file 1: Appendix H) during an eligible study visit with a non-antibiotic appropriate ARI diagnosis (listed in Table 1). At all study sites, providers prescribe antibiotics through the EHR; handwritten prescriptions are not used. An office visit is eligible for inclusion in the outcome denominator if: 1) the patient was 18 years old or older, 2) the provider and practice site were enrolled in the study, 3) the visit occurred during the 18-month intervention period, and 4) the patient did not have a visit with any ARI diagnosis in the prior 30 days. Visits are excluded from the primary analysis when: 1) patients have certain medical co-morbidities that make ARI guidelines less likely to apply, 2) patients had concomitant visit diagnoses indicating a non-ARI possible bacterial infection, or 3) patients had concomitant visit diagnoses indicating potentially antibiotic appropriate ARI diagnoses or other ARI diagnoses suggestive of a bacterial infection. Visits for which a provider recorded another condition that was not an ARI for which antibiotics might be indicated were also excluded from the analysis. The sets of diagnoses used to calculate the outcomes are listed in Additional file 1: Appendix E.

#### Secondary outcomes

We will examine oral antibiotic prescribing for the subgroup of qualifying visits with diagnoses for potentially antibiotic appropriate ARI diagnoses (acute sinusitis and acute pharyngitis; Table 1); with the same additional inclusion and exclusion criteria above.

We will also examine the antibiotic prescribing rate for all potential ARI visits in aggregate: non-antibiotic appropriate ARI diagnoses, potentially antibiotic appropriate ARI diagnoses, and other ARI diagnoses of interest listed in Table 1. Visits are excluded from this secondary analysis when: 1) patients have medical co-morbidities listed in Additional file 2: Appendix E, or 2) patients had concomitant visit diagnoses indicating a non-ARI possible bacterial infection (Additional file 1: Appendix E). Using a differences-in-differences approach, we will determine whether each intervention is associated with changes in the proportion of all antibiotics prescribed for these diagnostic categories, as compared to a pre-intervention period.

We will examine individual provider’s change in prescribing for non-antibiotic appropriate ARI diagnoses, potentially antibiotic appropriate ARI diagnoses, and ARIs in aggregate. We will examine whether the effects of the intervention differ according to the following provider subgroups: baseline prescribing rates (higher or lower), provider gender, or health system affiliation.

#### Qualitative data

For clinicians randomized to the Accountable Justification arm, we will use qualitative methods to analyze the content of their written justifications for prescribing antibiotics to patients with ARIs. To do this, we will create a comprehensive codebook to classify each justification, grouping the codes into coherent themes (e.g., guideline-concordant, guideline-exception, guideline-nonconcordant). Two or more trained physician coders will use the codebook to classify each justification independently. After initial independent coding, all cases of disagreements between coders will be resolved by discussion. Using the final codings, we will examine the distribution of justification codes in conjunction with clinicians’ antibiotic prescribing rates and quantify the extent to which persistent ARI antibiotic prescribing among Accountable Justifications clinicians is well-justified (i.e., prescribing that is either concordant with guidelines or for patients who are excluded from guidelines).

### Statistical analysis plan

In our primary model, we will test the impact of each of the 3 interventions and their interactions with each other. That is we will estimate the random effects model that an antibiotic will be prescribed at visit *i* by provider *j*.

$$\begin{array}{l} \mathrm{logit}\left\{P(y_{\mathrm{ij}}=1|\;x\right\}\\ \;=\beta_{0}+\beta_{1}x_{\mathrm{PC}}+\beta_{2}x_{\mathrm{AJ}}+\beta_{3}x_{\mathrm{SA}}+\beta_{4}x_{\mathrm{PC}}x_{\mathrm{AJ}}\\ \;+\beta_{5}x_{\mathrm{PC}}x_{\mathrm{SA}}+\beta_{6}x_{\mathrm{SA}}x_{\mathrm{AJ}}\\ \;+\beta_{7}x_{\mathrm{PC}}x_{\mathrm{SA}}x_{\mathrm{AJ}}+\epsilon_{j} \end{array}$$

Where PC is Peer Comparison, AJ is Accountable Justifications, and SA is Suggested Alternatives.

### Safety assessment plan

A Data Safety and Monitoring Board has been established. The board is composed of three physician experts in acute respiratory infection care, and meets biannually throughout the duration of the study to review patient safety and adverse events. Following each meeting, the board makes recommendations to the local IRBs as to whether the study should continue or if changes to the protocol are needed. Data for patients who have a return visit to a study clinic within 30 days of an eligible study visit with a diagnosis that could represent a serious complication of an untreated bacterial infection (e.g. acute rheumatic fever, head and neck abscess, intracranial abscess, Lemierre syndrome, mastoiditis, meningitis, pneumonia, sepsis, etc.) will be extracted from study site EHRs and reported to the Board.

### Power calculation

Using the correction for inter-cluster correlation from Kish, [44] we estimated the power of our study to detect a clinically significant difference between binary conditions. That is the sample size must be inflated by a factor of 1+ θ(*m*-1), where θ is the inter class correlation and *m* is the number of ARI observations per cluster. In our calculations we assumed an intra-clinic correlation of 0.055 6 and assumed a baseline antibiotic prescribing rate of 50%, an ARI visit rate of 15 visits per month for full time providers, and independence of treatments in the factorials design. We calculated the number of visits needed for an 80% chance to detect a clinically meaningful difference in antibiotic prescribing (7%). We assumed a 75% recruitment success rate for recruiting 376 eligible providers across 49 sites, resulting in 141 providers per study factor (282 providers total) and a one-sided α of 0.05. To achieve statistical power of 0.80 would require a total of 2252 visits at each factor level, or 4504 visits across all study conditions. Therefore, if each provider had a minimum of 16 antibiotic-inappropriate ARI visits over the course of the study, we would have sufficient power to detect a clinically significant effect.

## Results

### Characteristics of participating providers and clinical sites

We enrolled 269 of the 376 providers offered enrollment (72% participation rate). For Boston clinic participants, the mean age of providers is 49 years, 60% are female, and 85% are physicians. For Los Angeles clinic providers, the mean age is 45 years, are 61% female, and 71% are physicians. These providers worked at 49 primary care clinics affiliated with the 3 healthcare organizations (22 clinics from the Boston region and 27 clinics from the Los Angeles region). The results of the randomization procedure and the numbers of eligible providers who enrolled in the study are depicted in Table 2.

**Table 2:** Results of clinic randomization and provider enrollment

**Intervention**	**Randomized Clusters (n)**	**Providers enrolled (n) / providers eligible (n)**	**Percentage of eligible providers enrolled**	**Visits with non-antibiotic appropriate ARI diagnoses for enrolled providers in prior year**
No intervention	6	27 / 45	60%	1902
Accountable justifications	7	35 / 46	76%	1603
Suggested alternatives	6	44 / 57	77%	1658
Peer comparisons	6	33 / 37	89%	1141
Accountable justifications, suggested alternatives	6	36 / 49	73%	1592
Suggested alternatives, peer comparisons	6	36 / 58	62%	1861
Accountable justifications, peer comparisons	6	29 / 40	73%	1783
Accountable justifications, suggested alternatives, peer comparisons	6	29 / 44	66%	2358
Any accountable justifications	25	129 / 179	72%	7336
No accountable justifications	24	140 / 197	71%	6562
Any suggested alternatives	25	145 / 208	70%	7469
No suggested alternatives	24	124 / 168	74%	6429
Any peer comparisons	24	127 / 179	71%	7143
No peer comparisons	25	142 / 197	72%	6755

*ARI* acute respiratory infection.

Participating clinics represent diverse practice settings and patient populations. The Los Angeles clinics are predominantly federally qualified community health centers or other safety net clinics. The eastern Massachusetts clinics are academically affiliated and have high proportions of patients with commercial or Medicare insurance. The patient populations served by the eastern Massachusetts clinics are predominantly white (approximately 75%), while the majority of patients served by the Los Angeles clinics is Hispanic (84% at Alta Med and 71% at The Children’s Clinic), with a high proportion of residents living at or below 200% of the Federal Poverty Level.

## Discussion

Using a multi-factorial design, three interventions will be tested for their ability to alter inappropriate physician prescribing behavior: 1) Accountable Justifications triggered by guideline-discordant prescriptions that ask providers to provide their rationale for prescribing an antibiotic and include these rationales in the medical record; 2) Suggested Alternatives presented in EHR order sets containing guideline concordant treatment options for ARIs; and 3) Peer Comparisons communicated through emailed performance feedback reports that compare each provider’s own performance to his or her top-performing peers. These interventions build on two main preexisting quality improvement methods: computerized clinical decision support and performance feedback. The main innovation of the current study is that the study interventions are specifically designed to take advantage of irrational (but predictable) behavior patterns common to most human beings—including health care providers. In doing so, we hope to design interventions that will lead to greater provider behavior change than what has been observed in prior studies.

### Anticipated challenges and limitations

This study is being conducted in diverse practices that collectively use 3 different EHRs. This is both a limitation and a strength. Due to their adaptation to each EHR, the interventions differ somewhat across the different sites. The degree to which these differences might influence the effectiveness of the interventions is unknown, and we will examine whether the effects of each intervention are modified by health system. Adapting the study interventions to 3 different EHRs is a strength in that the findings may be generalizable to a wider range of practice settings than would findings from a study conducted within one provider network using a single information system.

## Abbreviations

ARI: Acute respiratory infection; BEARI: Behavioral economics to improve the treatment of acute respiratory infections; EHR: Electronic health record; IRB: Institutional review board.

## Competing interests

None of the authors have any competing interests relevant to this study.

## Authors’ contributions

JND, CRF, DM and MWF conceived the study. JND, SDP, MWF, DM, JAL, CRF, NJG, PS, and TKK participated in the design and/or implementation of study procedures. SDP, DM, TKK, and JND drafted portions of the manuscript. JND, DM, MWF, JAL, CRF, NJG, PS, TKK, SDP participated in critical revision of the manuscript for important intellectual content. All authors read and approved the final manuscript.

## Pre-publication history

The pre-publication history for this paper can be accessed here:

http://www.biomedcentral.com/1471-2334/13/290/prepub

## Supplementary Material

Additional file 1 **List of Supplemental Materials.** Appendix A: Survey and sample educational module at start of study. Appendix B: Post-study survey. Appendix C: Details of development and customization that was required at each site. Appendix D: Example of Accountable Justification decision support. Appendix F: Example of Suggested Alternatives order set. Appendix G: Sample Peer Comparison emails to providers. Appendix H: Oral antibiotics included in outcome measurements. Click here for file

Additional file 2 Appendix E New DX code set definitions. Click here for file
