# Supplementary material for: Use of behavioral economics and social psychology to improve treatment of acute respiratory infections (BEARI): rationale and design of a cluster randomized controlled trial [1RC4AG039115-01] - study protocol and baseline practice and provider characteristics
Source: BMC Infect Dis. 2013 Jun 27;13:290. doi: 10.1186/1471-2334-13-290 (PMC3701464; doi:10.1186/1471-2334-13-290)
Supplement: Additional file 1 — List of Supplemental Materials. Appendix A: Survey and sample educational module at start of study. Appendix B: Post-study survey. Appendix C: Details of development and customization that was required at each site. Appendix D: Example of Accountable Justification decision support. Appendix F: Example of Suggested Alternatives order set. Appendix G: Sample Peer Comparison emails to providers. Appendix H: Oral antibiotics included in outcome measurements. [file 1471-2334-13-290-S1.docx]

**List of Supplemental Materials**

**Appendix A: Survey and sample educational module at start of study**

**Appendix B: Post-study survey**

**Appendix C: Details of development and customization that was required at each site**

**Appendix D: Example of Accountable Justification decision support**

**Appendix E: Diagnosis code sets used in outcome assessments and clinical decision support**

**Appendix F: Example of Suggested Alternatives order set**

**Appendix G: Sample Peer Comparison emails to providers**

**Appendix H: Oral antibiotics included in outcome measurements**

**Appendix A: Survey and sample educational module at start of study**

**Online Survey**

| THE ONLINE SURVEY IS INTENDED TO (1) ELICIT INFORMATION FROM PROVIDERS (2) MONITOR IF “EDUCATON” INFLUENCES RESPONSES TO QUESTIONS ABOUT TREATMENT PREFERENCES. RESPONDENTS WILL HAVE THE OPPORTUNITY TO CHANGE THEIR ‘FINAL’ ANSWERS AT ANY TIME IN THE SURVEY. WE WILL RECORD ALL ANSWERS AND LOG CLICKS ON INFORMATIONAL LINKS PROVIDED. |
| --- |

Basic information about your clinical background.

1. When did you start working at [*name of clinic*]? (<1 year ago, 1-2 years ago, 3-5 years ago, 5-10 years ago, >10 years ago)
2. When did you finish your clinical training as a physician (i.e., your internship, residency, or fellowship—the one you most recently completed)? (<2 years ago, 2-5 years ago, 5-10 years ago, 10-20 years ago, >20 years ago)
3. What is your clinical specialty? (internal medicine, family practice, general practice, pediatrics, other)

Information about the electronic health record (EHR) used at your clinic.

1. How would you rate your overall level of satisfaction with the electronic health record (EHR) used at your clinic?
   (1= Very unsatisfied , 5=Very satisfied)
2. Thinking about your workflow during an office visit with a patient, how often do you enter at least 1 diagnosis for the visit into the EHR while you are still seeing the patient?
   1. Always
   2. Usually
   3. Sometimes
   4. Rarely
   5. Never
   6. Not applicable: The EHR does not offer a way to enter a diagnosis (or diagnoses) that correspond to the visit.

Quality improvement efforts.

1. Within the past year, have you received any feedback—positive or negative—from your clinic about the quality of care you provide to patients (for any kind of care)?
   1. Yes, positive feedback only
   2. Yes, both positive and negative feedback
   3. Yes, negative feedback only
   4. No, did not receive any feedback at all
   5. Unsure / Can’t Remember
2. [If yes to previous] Based on the feedback you received, did you make any changes to the way you deliver medical care?
   1. Yes, made 1 or more changes
   2. No, made no changes
   3. Unsure / Can’t Remember
3. In the past year, did you attend any medical educational sessions? *Note: “Medical education sessions” include sessions that yielded credit towards maintenance of certification (e.g., CME) and less formal sessions that did not yield such credit.*
   1. Yes
   2. No
   3. Unsure / Can’t Remember
4. [If yes to question 8] Based on the information you received in any of these educational sessions, did you make any changes to the way you deliver medical care?
   1. Yes, made 1 or more changes
   2. No, made no changes
   3. Unsure / Can’t Remember
5. [If yes to question 8] Did any of the educational sessions you attended cover the office-based treatment of acute respiratory infections (e.g., viral URIs, pharyngitis, bronchitis)?
   1. Yes
   2. No
   3. Unsure / Can’t Remember
6. [If yes to question 8] Did any of the educational sessions you attended cover the office-based treatment of acute low back pain?
   1. Yes
   2. No
   3. Unsure / Can’t Remember
7. Based on your general experience as a clinician, please indicate how much you agree or disagree with the following statements:
8. Continuing education is an effective way to improve the quality of care (1 = Strongly agree, 2 = Agree, 3 = Neither Agree nor Disagree, 4 = Disagree, 5 = Strongly Disagree)
9. Auditing physicians’ clinical performance and providing performance feedback is an effective way to improve the quality of care (1 = Strongly agree, 2 = Agree, 3 = Neither Agree nor Disagree, 4 = Disagree, 5 = Strongly Disagree)
10. Electronic decision support tools (e.g., “pop up” reminders in your EHR) are an effective way to improve the quality of care (1 = Strongly agree, 2 = Agree, 3 = Neither Agree nor Disagree, 4 = Disagree, 5 = Strongly Disagree)
11. Condition-specific, streamlined electronic order sets are an effective way to improve the quality of care (1 = Strongly agree, 2 = Agree, 3 = Neither Agree nor Disagree, 4 = Disagree, 5 = Strongly Disagree)

Your assessment of clinical guidelines.

| 1. Please indicate your level of knowledge about the following clinical guidelines. | [Know this guideline in detail / Know this guideline in general, but not every detail / Not familiar with this guideline] |
| --- | --- |
| *Screening* |  |
| Guidelines for colorectal cancer screening (USPSTF guideline: http://www.uspreventiveservicestaskforce.org/uspstf/uspscolo.htm) |  |
| Guidelines for breast cancer screening (USPSTF guideline: http://www.uspreventiveservicestaskforce.org/uspstf/uspsbrca.htm) |  |
| Guidelines for cervical cancer screening (USPSTF guideline: http://www.uspreventiveservicestaskforce.org/uspstf/uspscerv.htm) |  |
| *Chronic disease care* |  |
| Guidelines for the care of diabetes mellitus (ADA guideline: http://www.guideline.gov/content.aspx?id=15687) |  |
| Guidelines for lipid and cholesterol management (ATP III guidelines: http://circ.ahajournals.org/cgi/reprint/106/25/3143) |  |
| *Acute care* |  |
| Guidelines for antibiotic use in non-specific upper respiratory infections (CDC guidelines: http://www.annals.org/content/134/6/490.abstract) |  |
| Guidelines for imaging in acute low back pain (ACP/APS guidelines: http://www.annals.org/content/147/7/478.full.pdf+html) |  |

| 1. **In the grid below, please estimate the AVERAGE time allocated to you and amount of time you feel would be needed to provide high quality care for your patients.** *(please check one box)* | | |
| --- | --- | --- |
| *Visit type* | Time *allocated* | Time *needed* |
| 1. Complete Physical/Consultation | _______minutes | _______minutes |
| 1. Routine Follow-up Visits | _______minutes | _______minutes |
| 1. Urgent Care Visits (in general) | _______minutes | _______minutes |
| 1. Urgent Care Visits for acute respiratory infections | _______minutes | _______minutes |

| 1. **Which best describes the atmosphere in your office?** *(please check one box)* | Calm, orderly |  | Busy, but reasonable |  | Hectic, chaotic |
| --- | --- | --- | --- | --- | --- |
|  | □_1_ | □_2_ | □_3_ | □_4_ | □_5_ |

| 1. **Please indicate how much you agree or disagree with the following statement.** *(please check one box)* | Strongly disagree | Disagree | Neither agree nor disagree | Agree | Strongly Agree |
| --- | --- | --- | --- | --- | --- |
| Overall, I am satisfied with my current job | □_1_ | □_2_ | □_3_ | □_4_ | □_5_ |

**Educational Module**

**Key: AJ = Accountable Justifications, PC = Peer Comparison, SA = Suggested Alternatives.**

Guidelines for treating non-specific upper respiratory infections (URIs) in adults

- Definition
  - Acute infection in which sinus, pharyngeal, and lower airway symptoms, although frequently present, are not prominent
  - Also known as “the common cold”
- Causes
  - If systemic symptoms (e.g., myalgias, malaise) are prominent: influenza and parainfluenza infection
  - If systemic symptoms are less prominent: rhinoviruses, coronaviruses, adenoviruses, enteroviruses, and respiratory syncytial virus
- Diagnosis
  - Symptoms may include cough, sore throat, runny nose, nasal congestion, headache, low grade fever, facial pressure, sneezing
  - Purulent secretions from nares or throat do NOT indicate the presence of bacterial infection
- Course of illness
  - Duration of symptoms is usually 7-10 days.
- Guideline-consistent treatments
  - *These guidelines apply to immunocompetent adults without complicating comorbid conditions, such as chronic lung or heart disease*
  - Treat with decongestants, cough suppressants, and/or analgesics/antipyretics. For some patients, albuterol may also be appropriate.
  - Patient education: fluids, rest, salt water gargle.
  - Some patients may need a work excuse letter.
  - Antibiotics are not indicated.
  - (CDC recommendation link here)

*Response tasks (for PC-/AJ-/SA- subjects)*

- Thinking about the patients you see in clinic for non-specific URIs, how often does the guideline-based recommendation against prescribing antibiotics apply? [0-20% / 21-40% / 41-60% / 61-80% / 81-100% of patients / DK]
- How frequently do you prescribe antibiotics to your patients for the treatment of non-specific URIs? [0-20% / 21-40% / 41-60% / 61-80% / 81-100% of visits / DK]
- How frequently do you think other clinicians in your practice prescribe antibiotics for the treatment of non-specific URIs? [0-20% / 21-40% / 41-60% / 61-80% / 81-100% of visits/ DK]
- In some online studies, a small number of participants do not pay close attention to all of the items they are answering. To indicate that you are paying close attention, please do not mark any of the choices for the following question: How frequently do you think physicians prescribe decongestants for non-specific URIs? [0-20% / 21-40% / 41-60% / 61-80% / 81-100% of visits / DK]"

*Additional statements (for PC+ subjects)*

*[Injunctive norm] These guidelines for treating non-specific URIs have been endorsed by the American Academy of Family Physicians, the American College of Physicians, the Infectious Diseases Society of America, and the Centers for Disease Control and Prevention.*

*Response task additions (for PC+ subjects)*

- None (same as PC-)

*Intervention summary (for PC+ subjects)*

- During the study, you will receive regular updates on your own rate of antibiotic prescribing for patients who have non-specific URIs. As a demonstration of achievable performance, these updates will also include the antibiotic prescribing rate achieved by the 10% of physicians in [name of clinic] whose prescribing is most guideline-concordant.

*Additional statements (for AJ+ subjects)*

*[Importance of justification] Guidelines are intended to help clinicians treat the majority of their patients. However, there can be clinical reasons why a guideline might not apply to a particular patient. When there is a good clinical reason, a physician might justifiably choose not to follow a guideline.*

*If a physician decides that there is a clinically justifiable reason to prescribe antibiotics to a patient with a non-specific URI, he or she should have a clear sense of what this reason is. He or she should be able to state the justification for not following the guideline.*

*Response task additions (for AJ+ subjects)*

- To what extent do you agree with these overall guidelines for treating non-specific URIs? [completely agree to completely disagree]
- To what extent do you agree that antibiotics are not indicated for the treatment of non-specific URIs? [completely agree to completely disagree]
- (Other response tasks same as AJ-)…

*Intervention summary (for AJ+ subjects)*

- During the study, if you prescribe antibiotics to patient who you are seeing for a non-specific URI, you will be asked to supply a brief written justification for prescribing antibiotics. The justification that you write will be entered in the patient’s medical record. If you do not write a justification, the phrase “No justification for prescribing antibiotics was given” will appear in your encounter note.

*Additional statements (for SA+ subjects)*

*[Reminder about alternatives to antibiotics] For non-specific URIs, patients most often want a diagnosis and relief from symptoms; only a minority want antibiotics. Instead of antibiotics, you can prescribe medications that treat congestion, cough, sore throat, and general aches and pains to provide symptomatic relief. In most of these categories, there are both over-the-counter (OTC) and prescription options.*

*Even for OTC medications, writing a prescription can help your patients. In addition to serving as a reminder, writing a prescription will allow your patients to use their Flexible Savings Accounts (FSAs). Without your prescription, patients will be unable to use their FSAs to buy OTC medications.*

*In addition to medications, you can give patients educational materials that provide information and reassurance. You can use these materials as non-antibiotic treatments that address patients’ most common concerns.*

*Response task additions (for SA+ subjects)*

- None (same as SA-)

*Intervention summary (for SA+ subjects)*

- During the study, when you prescribe a medication to a patient who you diagnose with non-specific URI, you will be shown a list of non-antibiotic alternative prescriptions and symptomatic treatments. You will be able to select from among these treatment options, and corresponding prescriptions will be generated. You will also be able to select patient educational materials that will be printed for the patient you are seeing.
- Here is the list of non-antibiotic treatments that will be offered to you. If you want to prescribe a medication that does not appear on the list (including an antibiotic), you will be able to write this prescription as usual by closing the list. [show SA list below]

Guidelines for treating acute sinusitis/rhinosinusitis in adults

- Definition
  - “Sinusitis” refers to inflammation of the mucosa of the paranasal sinuses. Because inflammation of the nasal mucosa always accompanies sinusitis, “rhinosinusitis” has become the preferred term.
  - Rhinosinusitis is acute when of duration less than 4 weeks
- Causes
  - Most cases of acute rhinosinusitis diagnosed in ambulatory care are caused by uncomplicated viral upper respiratory tract infections.
  - Acute bacterial rhinosinusitis is usually a secondary infection resulting from sinus obstruction or impairment of mucus clearance mechanisms caused by an acute viral upper respiratory tract infection.
- Diagnosis
  - Patients with rhinosinusitis symptoms that last less than 7 days are unlikely to have bacterial infection, although rarely some patients with acute bacterial rhinosinusitis present with dramatic symptoms of severe unilateral maxillary pain, swelling, and fever.
  - The clinical diagnosis of acute bacterial rhinosinusitis should be reserved for patients with rhinosinusitis symptoms lasting 7 days or more and who have maxillary pain or tenderness in the face or teeth (especially when unilateral) and purulent nasal secretions.
- Course of illness
  - Acute rhinosinusitis resolves without antibiotic treatment in most cases.
  - Duration of symptoms is usually 7-10 days, but longer duration alone does not reliably indicate bacterial etiology.
- Guideline-consistent treatments
  - *These guidelines apply to adults who are not immunocompromised*
  - Treat with topical and systemic decongestants and/or analgesics/antipyretics.
  - Sinus radiography is not recommended.
  - Antibiotic therapy should be reserved for patients with moderately severe symptoms who meet the criteria for the clinical diagnosis of acute bacterial rhinosinusitis and for those with severe rhinosinusitis symptoms—especially those with unilateral facial pain—regardless of duration of illness.
    - For initial antibiotic treatment, use the most narrow-spectrum agent active against the likely pathogens, Streptococcus pneumoniae and Haemophilus influenzae: amoxicillin, doxycycline, and trimethoprim-sulfamethoxazole.
  - (CDC recommendation link here)

*Response tasks (for PC-/AJ-/SA- subjects)*

- Thinking about the patients you see in clinic for acute sinusitis/rhinosinusitis, how often do the guideline-based recommendations for prescribing antibiotics apply? [0-20% / 21-40% / 41-60% / 61-80% / 81-100% of patients / DK]
- How frequently do you prescribe antibiotics to your patients for the treatment of acute sinusitis/rhinosinusitis? [0-20% / 21-40% / 41-60% / 61-80% / 81-100% of visits / DK]
- How frequently do you think other clinicians in your practice prescribe antibiotics for the treatment of acute sinusitis/rhinosinusitis ? [0-20% / 21-40% / 41-60% / 61-80% / 81-100% of visits / DK]

*Additional statements (for PC+ subjects)*

*[Injunctive norm] These guidelines for treating acute sinusitis/rhinosinusitis have been endorsed by the American Academy of Family Physicians, the American College of Physicians, the Infectious Diseases Society of America, and the Centers for Disease Control and Prevention.*

*Response task addition (for PC+ subjects)*

- None (same as PC-)

*Intervention summary (for PC+ subjects)*

- During the study, you will receive regular updates on your own rate of antibiotic prescribing for patients who have acute sinusitis/rhinosinusitis. As a demonstration of achievable performance, these updates will also include the antibiotic prescribing rate achieved by the 10% of physicians in [name of clinic] whose prescribing rates are lowest.

*Additional statements (for PC+ subjects)*

*[Injunctive norm] These guidelines for treating acute sinusitis/rhinosinusitis have been endorsed by the American Academy of Family Physicians, the American College of Physicians, the Infectious Diseases Society of America, and the Centers for Disease Control and Prevention.*

*Response task addition (for PC+ subjects)*

- None (same as PC-)

*Intervention summary (for PC+ subjects)*

- During the study, you will receive regular updates on your own rate of antibiotic prescribing for patients who have acute sinusitis/rhinosinusitis. As a demonstration of achievable performance, these updates will also include the antibiotic prescribing rate achieved by the 10% of physicians in [name of clinic] whose prescribing rates are lowest.

*Additional statements (for SA+ subjects)*

*[Reminder about alternatives to antibiotics] Regardless of whether antibiotics are prescribed for acute sinusitis/rhinosinusitis, decongestants may enable drainage of sinus secretions, and analgesics/antipyretics can provide symptomatic relief. You can also prescribe these medications instead of antibiotics when antibiotics are not indicated. There are both over-the-counter (OTC) and prescription-only options for decongestants and analgesics/antipyretics.*

*Even for OTC medications, writing a prescription can help your patients. In addition to serving as a reminder, writing a prescription will allow your patients to use their Flexible Savings Accounts (FSAs). Without your prescription, patients will be unable to use their FSAs to buy OTC medications.*

*In addition to medications, you can give patients educational materials that provide information and reassurance. You can use these materials as non-antibiotic treatments that address patients’ most common concerns.*

*Response task addition (for SA+ subjects)*

- None (same as SA-)

*Intervention summary (for SA+ subjects)*

- During the study, when you prescribe a medication to a patient who might have acute sinusitis/rhinosinusitis, you will be shown a list of non-antibiotic alternative prescriptions and symptomatic treatments. You will be able to select from among these treatment options, and corresponding prescriptions will be generated. You will also be able to select patient educational materials that will be printed for the patient you are seeing.
- Here is the list of non-antibiotic treatments that will be offered to you. If you want to prescribe a medication that does not appear on the list (including an antibiotic), you will be able to write this prescription as usual by closing the list. [show SA list below]

*Additional statements (for AJ+ subjects)*

*[Importance of justification] Guidelines are intended to help clinicians treat the majority of their patients. However, there can be clinical reasons why a guideline might not apply to a particular patient. When there is a good clinical reason, a physician might justifiably choose not to follow a guideline.*

*For acute sinusitis/rhinosinusitis, antibiotics may be prescribed in guideline-consistent or guideline-inconsistent ways. In either case, a physician who decides that there is a clinically justifiable or guideline-consistent reason to prescribe antibiotics to a patient with acute sinusitis/rhinosinusitis should have a clear sense of what this reason is. He or she should be able to state this justification or explain how the guideline was followed.*

*Response task additions (for AJ+ subjects)*

- To what extent do you agree with these overall guidelines for treating acute sinusitis/rhinosinusitis? [completely agree to completely disagree]
- To what extent do you agree that antibiotics are not indicated for the treatment of acute sinusitis/rhinosinusitis of <7 days’ duration in most patients? [completely agree to completely disagree]
- (Other response tasks same as AJ-)…

*Intervention summary (for AJ+ subjects)*

- During the study, if you prescribe antibiotics to patient who you are seeing for acute sinusitis/rhinosinusitis, you will be asked to supply a brief written justification for prescribing antibiotics. The justification that you write will be entered in the patient’s medical record. If you do not write a justification, the phrase “No justification for prescribing antibiotics was given” will appear in your encounter note.

Guidelines for treating acute pharyngitis in adults

- Definition
  - Cases of acute sore throat of <7 days duration in which specific rarer causes of sore throat (e.g., gonococcus, diphtheria, epiglottitis, Ludwig angina, acute HIV infection, retropharygeal abscess, trauma) are not present.
- Causes
  - Viruses are the most common cause
  - Group A beta-hemolytic streptococcus (GABHS) is the cause of approximately 10% of adult cases of pharyngitis
- Treatment principles
  - The major reason to treat adults with GABHS pharyngitis is symptomatic relief. In patients with GABHS, antibiotic therapy instituted within 2 to 3 days of symptom onset shorten the duration of symptoms by 1 to 2 days.
  - Antibiotics do not hasten symptomatic improvement in patients without GABHS.
  - Complications of GABHS pharyngitis are rare in immunocompetent adults.
    - In cases of GABHS pharyngitis, there is no evidence that antibiotics reduce the incidence of acute glomerulonephritis.
    - In the United States, the incidence of acute rheumatic fever fell by a factor of 60 between 1965 and 1994. In order to prevent one case of acute rheumatic fever, current estimates of the number of patients with GABHS that would need treatment with antibiotics range from 3000-4000 patients.
  - There is no evidence that the use of antibiotics reduces the spread of GABHS among non-institutionalized adult patients. The pre-symptomatic incubation period for GABHS is 2-5 days, during which patients may expose their close contacts.
- Diagnosis
  - The most reliable predictors of GABHS pharyngitis are the Centor criteria:
    - Tonsillar exudates
    - Tender anterior cervical lymphadenopathy or lymphadenitis
    - Absence of cough
    - History of fever
  - The positive predictive value of the presence of 3-4 Centor criteria is 40-60% for GABHS pharyngitis.
  - The negative predictive value of 0, 1, or 2 Centor criteria is ~80% for the absence of GABHS pharyngitis.
  - Rapid antigen tests for GABHS can be combined with the Centor criteria
  - Throat cultures are not recommended for routine use in cases of acute pharyngitis in adults
- Course of illness
  - For viral acute pharyngitis, the duration of symptoms is usually 5 to 7 days.
- Guideline-consistent treatments (for immunocompetent adults without complicated comorbid conditions, such as chronic lung or heart disease, or history of rheumatic fever, in the absence of known local GAHBS outbreaks)
  - Treat all patient with analgesics/antipyretics. For patients with co-occurring nasal congestion and post-nasal drip, decongestants may also be helpful.
  - Patient education: fluids, rest, salt water gargle.
  - Some patients may need a work excuse letter
  - Use of antibiotics: three strategies are acceptable
    1. Test patients (rapid antigen) with 2, 3, or 4 Centor criteria, and limit antibiotic therapy to patients with positive test results.
    2. Test patients (rapid antigen) with 2 or 3 Centor criteria test, and limit antibiotic therapy to patients with positive test results or patients with four criteria.
    3. Do not use any diagnostic tests, and limit antibiotic therapy to patients with 3 or 4 Centor criteria.
  - If antibiotics are used, guideline-consistent options are:
    1. a single dose of intramuscular penicillin G benzathine (1.2 MU for adults)
    2. standard penicillin VK, 500 mg orally twice or three times daily for 10 days
    3. in penicillin-allergic patients, use erythromycin 500 mg twice daily
  - (CDC recommendation link here)

*Response tasks (for PC-/AJ-/SA- subjects)*

- Thinking about the patients you see in clinic for acute pharyngitis, how often do the guideline-based recommendations for prescribing antibiotics apply? [0-20% / 21-40% / 41-60% / 61-80% / 81-100% of patients / DK]
- How frequently do you prescribe antibiotics to your patients for the treatment of acute pharyngitis when clinical and/or testing criteria are not met? [0-20% / 21-40% / 41-60% / 61-80% / 81-100% of visits / DK]
- How frequently do you think other clinicians in your practice prescribe antibiotics for the treatment of acute pharyngitis when clinical and/or testing criteria are not met? [0-20% / 21-40% / 41-60% / 61-80% / 81-100% of visits / DK]

*Additional statements (for PC+ subjects)*

*[Injunctive norm] These guidelines for treating acute pharyngitis have been endorsed by the American Academy of Family Physicians, the American College of Physicians, and the Centers for Disease Control and Prevention.*

*Response task additions (for PC+ subjects)*

- None (same as PC-)

*Intervention summary (for PC+ subjects)*

- During the study, you will receive regular updates on your own rate of antibiotic prescribing for patients who have non-GABHS acute pharyngitis. As a demonstration of achievable performance, these updates will also include the antibiotic prescribing rate achieved by the 10% of physicians in [name of clinic] whose prescribing rates are lowest for non-GABHS acute pharyngitis.

*Additional statements (for AJ+ subjects)*

*[Importance of justification] Guidelines are intended to help clinicians treat the majority of their patients. However, there can be clinical reasons why a guideline might not apply to a particular patient. When there is a good clinical reason, a physician might justifiably choose not to follow a guideline.*

*For acute pharyngitis, antibiotics may be prescribed in guideline-consistent or guideline-inconsistent ways. In either case, a physician who decides that there is a clinically justifiable or guideline-consistent reason to prescribe antibiotics to an adult patient with acute pharyngitis should have a clear sense of what this reason is. He or she should be able to state this justification or explain how the guideline was followed.*

*Response task additions (for AJ+ subjects)*

- To what extent do you agree with these guidelines for treating acute pharyngitis in adults? [completely agree to completely disagree]
- To what extent do you agree that antibiotics are not indicated for the treatment of acute pharyngitis in healthy patients when clinical and/or testing criteria are not met? [completely agree to completely disagree]
- (Other response tasks same as AJ-)…

*Intervention summary (for AJ+ subjects)*

- During the study, if you prescribe antibiotics to patient who you are seeing for acute pharyngitis, you will be asked to supply a brief written justification for prescribing antibiotics. The justification that you write will be entered in the patient’s medical record. If you do not write a justification, the phrase “No justification for prescribing antibiotics was given” will appear in your encounter note.

*Additional statements (for SA+ subjects)*

*[Reminder about alternatives to antibiotics] Regardless of whether antibiotics are prescribed for acute pharyngitis, analgesics/antipyretics can provide symptomatic relief. You can also prescribe these medications instead of antibiotics when antibiotics are not indicated. There are both over-the-counter (OTC) and prescription-only options for analgesics/antipyretics.*

*Even for OTC medications, writing a prescription can help your patients. In addition to serving as a reminder, writing a prescription will allow your patients to use their Flexible Savings Accounts (FSAs). Without your prescription, patients will be unable to use their FSAs to buy OTC medications.*

*In addition to medications, you can give patients educational materials that provide information and reassurance. You can use these materials as non-antibiotic treatments that address patients’ most common concerns.*

*Response task additions (for SA+ subjects)*

- None (same as SA-)

*Intervention summary (for SA+ subjects)*

- During the study, when you prescribe a medication to a patient who might have acute pharyngitis, you will be shown a list of non-antibiotic alternative prescriptions and symptomatic treatments. You will be able to select from among these treatment options, and corresponding prescriptions will be generated. You will also be able to select patient educational materials that will be printed for the patient you are seeing.
- Here is the list of non-antibiotic treatments that will be offered to you. If you want to prescribe a medication that does not appear on the list (including an antibiotic), you will be able to write this prescription as usual by closing the list. [show SA list below]

Guidelines for treating acute bronchitis in adults

- Definition
  - “Acute bronchitis” refers to an acute respiratory tract infection of duration <3 weeks in which cough, with or without phlegm, is a predominant feature.
- Causes
  - Over 90% of acute bronchitis cases are not caused by bacteria.
  - Viruses most frequently associated with acute bronchitis include influenza B, influenza A, parainfluenza 3, respiratory syncytial virus, coronaviruses, adenoviruses, and rhinoviruses.
- Diagnosis
  - A diagnosis of acute bronchitis requires the exclusion of pneumonia. Physical examination and chest X-rays, considered in the context of specific patient and epidemiologic circumstances, are common diagnostic steps.
  - When and coughing illness last longer than 3 weeks, previously undiagnosed asthma should be considered.
  - Purulent sputum does not distinguish bronchitis from pneumonia, indicate that bacterial are present, or mean that antibiotics are necessary.
- Course of illness
  - The average duration of cough for adults with uncomplicated acute bronchitis is 2-3 weeks.
- Guideline-consistent treatments (for immunocompetent adults without complicating comorbid conditions, such as chronic lung or heart disease)
  - Treatment with cough suppressants, analgesics/antipyretics, and inhaled albuterol may relieve symptoms but not shorten the duration of illness. Elimination of environmental cough triggers (for example, dust and dander) and vaporized air treatments (particularly in low-humidity environments) are also reasonable options.
  - Provide realistic expectations for the duration of the patient’s cough, which will typically last 10 to 14 days after the office visit.
  - Routine antibiotic treatment of uncomplicated acute bronchitis is not recommended, regardless of duration of cough.
  - The evidence supports antibiotic treatment of patients with uncomplicated acute bronchitis only when there is suspicion of pertussis (i.e., when there is a high probability of exposure—for example, during documented local outbreaks). Antibiotic treatment, which does not shorten the duration of symptoms if it is initiated 7 to 10 days after onset of illness, decreases shedding of the pathogen and spread of disease. Antibiotic treatment of suspected pertussis should always be accompanied by a diagnostic test for public health purposes.
  - (CDC recommendation link here)

*Response tasks (for PC-/AJ-/SA- subjects)*

- Thinking about the patients you see in clinic for uncomplicated acute bronchitis, how often does the guideline-based recommendation against prescribing antibiotics apply? [0-20% / 21-40% / 41-60% / 61-80% / 81-100% of patients / DK]
- How frequently do you prescribe antibiotics to your patients for the treatment of uncomplicated acute bronchitis? [0-20% / 21-40% / 41-60% / 61-80% / 81-100% of visits / DK]
- How frequently do you think other clinicians in your practice prescribe antibiotics for the treatment of uncomplicated acute bronchitis? [0-20% / 21-40% / 41-60% / 61-80% / 81-100% of visits / DK]

*Additional statements (for PC+ subjects)*

*[Injunctive norm] These guidelines for treating acute pharyngitis have been endorsed by the American Academy of Family Physicians, the American College of Physicians, the Infectious Diseases Society of America, and the Centers for Disease Control and Prevention.*

*Response task addition (for PC+ subjects)*

- None (same as PC-)

*Intervention summary (for PC+ subjects)*

- During the study, you will receive regular updates on your own rate of antibiotic prescribing for patients who have uncomplicated acute bronchitis. As a demonstration of achievable performance, these updates will also include the antibiotic prescribing rate achieved by the 10% of physicians in [name of clinic] whose prescribing rates are the lowest.

*Additional statements (for AJ+ subjects)*

*[Importance of justification] Guidelines are intended to help clinicians treat the majority of their patients. However, there can be clinical reasons why a guideline might not apply to a particular patient. When there is a good clinical reason, a physician might justifiably choose not to follow a guideline.*

*If a physician decides that there is a clinically justifiable reason to prescribe such antibiotics to a patient with acute bronchitis, he or she should have a clear sense of what this reason is. He or she should be able to state the justification for not following the guideline.*

*Response task modification (for AJ+ subjects)*

- To what extent do you agree with these overall guidelines for treating uncomplicated acute bronchitis? [completely agree to completely disagree]
- To what extent do you agree that antibiotics are not indicated for the treatment of uncomplicated acute bronchitis in healthy patients? [completely agree to completely disagree]
- (Other response tasks same as AJ-)…

*Intervention summary (for AJ+ subjects)*

- During the study, if you prescribe antibiotics to patient who you are seeing for acute bronchitis, you will be asked to supply a brief written justification for prescribing antibiotics. The justification that you write will be entered in the patient’s medical record. If you do not write a justification, the phrase “No justification for prescribing antibiotics was given” will appear in your encounter note.

*Additional statements (for SA+ subjects)*

*[Reminder about alternatives to antibiotics]. To provide symptomatic relief to patients with acute bronchitis, you can prescribe cough suppressants, analgesics/antipyretics, and inhaled albuterol. You can prescribe these medications instead of antibiotics when antibiotics are not indicated. There are both over-the-counter (OTC) and prescription-only options for cough suppressants and analgesics/antipyretics.*

*Even for OTC medications, writing a prescription can help your patients. In addition to serving as a reminder, writing a prescription will allow your patients to use their Flexible Savings Accounts (FSAs). Without your prescription, patients will be unable to use their FSAs to buy OTC medications.*

*In addition to medications, you can give patients educational materials that provide information and reassurance. You can use these materials as non-antibiotic treatments that address patients’ most common concerns.*

*Response task addition (for SA+ subjects)*

- None (same as SA-)

*Intervention summary (for SA+ subjects)*

- During the study, when you prescribe a medication to a patient who might have acute bronchitis, you will be shown a list of non-antibiotic alternative prescriptions and symptomatic treatments. You will be able to select from among these treatment options, and corresponding prescriptions will be generated. You will also be able to select patient educational materials that will be printed for the patient you are seeing.
- Here is the list of non-antibiotic treatments that will be offered to you. If you want to prescribe a medication that does not appear on the list (including an antibiotic), you will be able to write this prescription as usual by closing the list. [show SA list below]

**Appendix B: Post-study survey**

**BEARI EXIT SURVEY**

1) How would you rate your overall level of satisfaction with the electronic health record (EHR) used at your clinic?
(1= Very unsatisfied , 2=Unsatisfied, 3=Neither satisfied nor satisfied 4=Satisfied 5=Very satisfied)

a) How would you rate your overall satisfaction with the alerts and clinical decision support you received for patients with acute respiratory infections?
(1= Very unsatisfied , 2=Unsatisfied, 3=Neither satisfied nor satisfied 4=Satisfied 5=Very satisfied, 0= I didn’t receive alerts for acute respiratory infections.

b) How would you rate your overall level of satisfaction with the antibiotic over-prescription feedback e-mails that have been sent out?”
(1= Very unsatisfied , 2=Unsatisfied, 3=Neither satisfied nor satisfied 4=Satisfied 5=Very satisfied)

2) Based on your general experience as a clinician, please indicate how much you agree or disagree with the following statements:

i. Auditing physicians’ clinical performance and providing performance feedback is an effective way to improve the quality of care (1 = Strongly agree, 2 = Agree, 3 =Neither Agree nor Disagree, 4 = Disagree, 5 = Strongly Disagree)

ii. Electronic decision support tools (e.g., reminders and alerts in your EHR) are an effective way to improve the quality of care (1 = Strongly agree, 2 = Agree, 3 =Neither Agree nor Disagree, 4 = Disagree, 5 = Strongly Disagree)

iii. Condition-specific, streamlined electronic order sets are an effective way to improve the quality of care (1 = Strongly agree, 2 = Agree, 3 = Neither Agree nor Disagree, 4 = Disagree, 5 = Strongly Disagree)

3)

| 1. **In the grid below, please estimate the AVERAGE time allocated to you and amount of time you feel would be needed to provide high quality care for your patients.** *(please check one box)* | | |
| --- | --- | --- |
| *Visit type* | Time *allocated* | Time *needed* |
| 1. Complete Physical/Consultation | _______minutes | _______minutes |
| 1. Routine Follow-up Visits | _______minutes | _______minutes |
| 1. Urgent Care Visits (in general) | _______minutes | _______minutes |
| 1. Urgent Care Visits for acute respiratory infections | _______minutes | _______minutes |

| 1. **Which best describes the atmosphere in your office?** *(please check one box)* | Calm, orderly |  | Busy, but reasonable |  | Hectic, chaotic |
| --- | --- | --- | --- | --- | --- |
|  | □_1_ | □_2_ | □_3_ | □_4_ | □_5_ |

| 1. **Please indicate how much you agree or disagree with the following statement.** *(please check one box)* | Strongly disagree | Disagree | Neither agree nor disagree | Agree | Strongly Agree |
| --- | --- | --- | --- | --- | --- |
| Overall, I am satisfied with my current job | □_1_ | □_2_ | □_3_ | □_4_ | □_5_ |

| **4) Please indicate how much you agree or disagree with the following statements.** | Strongly Disagree | Disagree | Neither Agree nor Disagree | Agree | Strongly Agree |
| --- | --- | --- | --- | --- | --- |
| 1. I receive useful information about the quality of care I deliver | □_1_ | □_2_ | □_3_ | □_4_ | □_5_ |
| 1. When I receive a new report about the quality of care, it just makes me feel helpless | □_1_ | □_2_ | □_3_ | □_4_ | □_5_ |
| 1. My practice evaluates me in a way that is fair | □_1_ | □_2_ | □_3_ | □_4_ | □_5_ |

5) *Response tasks --* Non-specific upper respiratory infections
 Click here for a brief guideline review :
<http://www.annals.org/content/134/6/490.abstract>

- How frequently do you prescribe antibiotics to your patients for the treatment of non-specific URIs?
  [0-20% / 21-40% / 41-60% / 61-80% / 81-100% of visits / Don’t Know]
- How frequently do you think other clinicians in your practice prescribe antibiotics for the treatment of non-specific URIs?
   [0-20% / 21-40% / 41-60% / 61-80% / 81-100% of visits/ Don’t Know]
- How frequently do you think the top 10% of clinicians in your practice prescribe antibiotics for the treatment of non-specific URIs? [0-20% / 21-40% / 41-60% / 61-80% / 81-100% of visits/ Don’t Know]
- Realistically, for patients without chronic conditions, how low do you think a good doctor could get their antibiotic prescribing rate for non-specific URIs?

[0-20% / 21-40% / 41-60% / 61-80% / 81-100% of visits/ Don’t Know]

- In some online studies, a small number of participants do not pay close attention to all of the items they are answering. To indicate that you are paying close attention, please do not mark any of the choices for the following question: How frequently do you think physicians prescribe decongestants for non-specific URIs?
   [0-20% / 21-40% / 41-60% / 61-80% / 81-100% of visits / Don’t Know]

6) *Response tasks –Sinusitis* Click here for a brief guideline review:
<http://www.cdc.gov/getsmart/campaign-materials/info-sheets/adult-approp-summary.html>

- How frequently do you prescribe antibiotics to your patients for the treatment of acute sinusitis/rhinosinusitis?
   [0-20% / 21-40% / 41-60% / 61-80% / 81-100% of visits / Don’t Know]
- How frequently do you think other clinicians in your practice prescribe antibiotics for the treatment of acute sinusitis/rhinosinusitis?
  [0-20% / 21-40% / 41-60% / 61-80% / 81-100% of visits / Don’t Know]
- How frequently do you think the top 10% of clinicians in your practice prescribe antibiotics for the treatment of acute sinusitis/rhinosinusitis?
  [0-20% / 21-40% / 41-60% / 61-80% / 81-100% of visits / Don’t Know]
- Realistically, for patients without chronic conditions, how low do you think a good doctor could get their antibiotic prescribing rate for sinusitis?
  [0-20% / 21-40% / 41-60% / 61-80% / 81-100% of visits / Don’t Know]

7) *Response tasks--*Acute pharyngitis
 Click here for a brief guideline review:
<http://www.cdc.gov/getsmart/campaign-materials/info-sheets/adult-approp-summary.html>

- How frequently do you prescribe antibiotics to your patients for the treatment of acute pharyngitis when clinical and/or testing criteria are not met?
   [0-20% / 21-40% / 41-60% / 61-80% / 81-100% of visits / Don’t Know]
- How frequently do you think other clinicians in your practice prescribe antibiotics for the treatment of acute pharyngitis when clinical and/or testing criteria are not met?
  [0-20% / 21-40% / 41-60% / 61-80% / 81-100% of visits / Don’t Know]
- How frequently do you think the top 10% of clinicians in your practice prescribe antibiotics for the treatment of acute pharyngitis when clinical and/or testing criteria are not met?
  [0-20% / 21-40% / 41-60% / 61-80% / 81-100% of visits / Don’t Know]
- Realistically, for patients without chronic conditions, how low do you think a good doctor could get their antibiotic prescribing rate for acute pharyngitis?
  [0-20% / 21-40% / 41-60% / 61-80% / 81-100% of visits / Don’t Know]

8) *Response tasks*-- Acute bronchitis
Click here for a brief guideline review:
<http://www.cdc.gov/getsmart/campaign-materials/info-sheets/adult-approp-summary.html>

- How frequently do you prescribe antibiotics to your patients for the treatment of uncomplicated acute bronchitis?
  [0-20% / 21-40% / 41-60% / 61-80% / 81-100% of visits / Don’t Know]
- How frequently do you think other clinicians in your practice prescribe antibiotics for the treatment of uncomplicated acute bronchitis?
  [0-20% / 21-40% / 41-60% / 61-80% / 81-100% of visits / Don’t Know]
- How frequently do you think the top 10% of clinicians in your practice prescribe antibiotics for the treatment of uncomplicated acute bronchitis?
  [0-20% / 21-40% / 41-60% / 61-80% / 81-100% of visits / Don’t Know]
- Realistically, for patients without chronic conditions, how low do you think a good doctor could get their antibiotic prescribing rate for acute bronchitis?
  [0-20% / 21-40% / 41-60% / 61-80% / 81-100% of visits / Don’t Know]

9) At this point in time, how would you rate your overall level of satisfaction with the e-mails that you recently received about your antibiotic prescribing?
(1= Very unsatisfied , 2, 3, 4, 5=Very satisfied)

10) How useful did you find the program in improving antibiotic prescribing practices?
(1=Not at all , 2, 3, 4, 5=Very)

11) How useful did you find the peer comparison information regarding the top performers’ over-prescription rate?
(1=Not at all, 2, 3, 4, 5=Very)

12) Please read the following vignette and then answer the questions below.

*Acute Bronchitis*

A 27-year-old woman with no known underlying lung disease presents with a 10-day history of cough that is productive of yellow nonbloody sputum. Her symptoms began with nasal congestion and a sore throat, but these initial symptoms resolved after a few days. Her remaining symptom is the cough which is less productive than it was several days ago. She denies any known sick contacts. Her cough does not occur in long fits, and there is no post-tussive emesis. On physical examination she is not in respiratory distress, afebrile, and has normal vital signs. Lung exam is normal.

- - 1. The probability of a major benefit from prescribing erythromycinis: [enter a number from 0-100 here] %.
    2. The probability of a minor benefit from named antibiotic (e.g., the patient feels better a day or two sooner vs. not getting the antibiotic), is: [enter a number from 0-100 here] %.
    3. The probability of a minor harm from prescribing erythromycin (e.g., temporary diarrhea or yeast infection), is : [enter a number from 0-100 here] %.
    4. The probability of a major harm from prescribing erythromycin (e.g., serious drug reaction including anaphylaxis, cardiac arrhythmia) is: [enter a number from 0-100 here] %.
    5. What are the chances you would prescribe erythromycin? [enter a number from 0-100 here] %.
    6. Now imagine that the patient specifically requests an antibiotic.  What are the chances you would prescribe the antibiotic? [enter a number from 0-100 here] %.

13) Are you more frequently engaging patients in antibiotics discussions since the study started?
Yes ☐

No ☐

| **14) Please estimate the percentage of your patients in each of these categories:** |
| --- |

|  |  | *b.* Have complex or numerous medical problems | _____% |
| --- | --- | --- | --- |
|  |  | f. *c.* Have complex or numerous psycho-social problems | _____% |
|  |  | *d.* Are generally frustrating to deal with | _____% |
| *a.* Suffer from chronic pain | _____% | *e.* Have alcohol or other substance abuse disorders | _____% |

| 15) **Please indicate how much you agree or disagree with the following statements.** | Strongly disagree | Disagree | Neither agree nor disagree | Agree | Strongly Agree |
| --- | --- | --- | --- | --- | --- |
| 1. Many patients demand potentially unnecessary treatments | □_1_ | □_2_ | □_3_ | □_4_ | □_5_ |
| 1. Time pressures keep me from developing good patient relationships | □_1_ | □_2_ | □_3_ | □_4_ | □_5_ |
| 1. I am overwhelmed by the needs of my patients | □_1_ | □_2_ | □_3_ | □_4_ | □_5_ |

16) For a typical ARI patient, antibiotics are more likely to do harm (primarily diarrhea

and yeast infections) than to do good (by speeding recovery or preventing some kind of

bacterial complication).

Please rate your agreement from 1 (Low) to 10 (High). *

1 2 3 4 5 6 7 8 9 10

17) Inappropriate antibiotic prescribing to ARI patients is caused by patients' “demand” for antibiotics.

Please rate your agreement from 1 (Low) to 10 (High). *

1 2 3 4 5 6 7 8 910

18) Is inappropriate ARI prescribing caused by doctors having not enough time with patients?

Please rate your agreement from 1 (Low) to 10 (High). *

1 2 3 4 5 6 7 8 9 10

19) Do you generally support performance measurement and quality improvement for

doctors’ practices?

Please rate your agreement from 1 (Low) to 10 (High). *

1 2 3 4 5 6 7 8 910


**20-29) Conjoint Analysis Example:**

Consider each of the following Choice Pairs. For each separate Choice Pair indicate which treatment option is preferable to you. You may only indicate one choice per pair.

|  | CHOICE A | CHOICE B |
| --- | --- | --- |
| EHR Prescribing Default Screen | ON | OFF |
| Peer Performance Feedback | OFF | ON |
| Pay for performance | $12/month | $100/month |
| Additional ARI Therapy Explanation Time | 5 minutes per visit | 1 minute per visit |
|  |  |  |
| MY CHOICE | X |  |

We will now give you 10 choice pairs and ask you to indicate your choice preference for each pair sequentially. Please pick the one of the two alternatives that you think is better.

[Choice Pairs 1 through 10]

30) Please answer the following questions:

(a) A bat and a ball cost $1.10 in total. The bat costs $1.00 more than the ball.
How much does the ball cost? _____ cents

(b) If it takes 5 machines 5 minutes to make 5 widgets, how long would it take
100 machines to make 100 widgets? _____ minutes

(c) In a lake, there is a patch of lily pads. Every day, the patch doubles in size.
If it takes 48 days for the patch to cover the entire lake, how long would it
take for the patch to cover half of the lake? _____ days

**You have completed the exit survey. Thank you very much for your participation!**

**Appendix C : Details of development and customization that was required at each site**

| **Requirement/Feature** | **Longitudinal Medical Record** | **NextGen** | **EpicCare** |
| --- | --- | --- | --- |
| **Application of eMeasure exclusion criteria from problem list, past diagnoses, and concomitant diagnoses to suppress alerts** | Not Implemented | Typical customization of existing functionality | Typical customization of existing functionality |
| **Justification Text Inserted into a visible portion of the medical record** | Expert programming | Expert programming | Expert programming |
| **Default text “No justification Provided” populating medical record** | Expert programming | Expert programming | Expert programming |
| **Prompt for diagnosis entry if absent at time of prescription order** | Typical customization of existing functionality | Not applicable (normal workflow) | Typical customization of existing functionality |
| **Custom Design Order Sets** | Typical customization of existing functionality | Typical customization of existing functionality | Typical customization of existing functionality |
| **Diagnosis Triggered Order Sets** | Typical customization of existing functionality | Typical customization of existing functionality | Typical customization of existing functionality |
| **Mechanism to remove antibiotic prescription ordered but not yet signed** | Expert programming: Medication automatically cancelled with option to reinstate with checkbox | Alerts include a custom-programmed medication management interface to remove pending prescriptions | Alert includes a link allowing user to rapidly navigate user back to the normal order interface to modify pending orders |
| **Accountable justifications alert stops showing** | Expert programming | After alert has been acknowledged or times out | After alert has been acknowledged or bypassed |
| **Alerts reappear if bypassed?** | Alerts appear for all antibiotic prescriptions at which point clinician indicates it is for an ARI. | Upon encounter sign out if antibiotic has been ordered. | Upon encounter sign out if antibiotic has been ordered. |
| **Development and testing** | 5 months | 7 months | 10 months |
|  | | | |

**Appendix D: Example of Accountable Justification decision support**

**
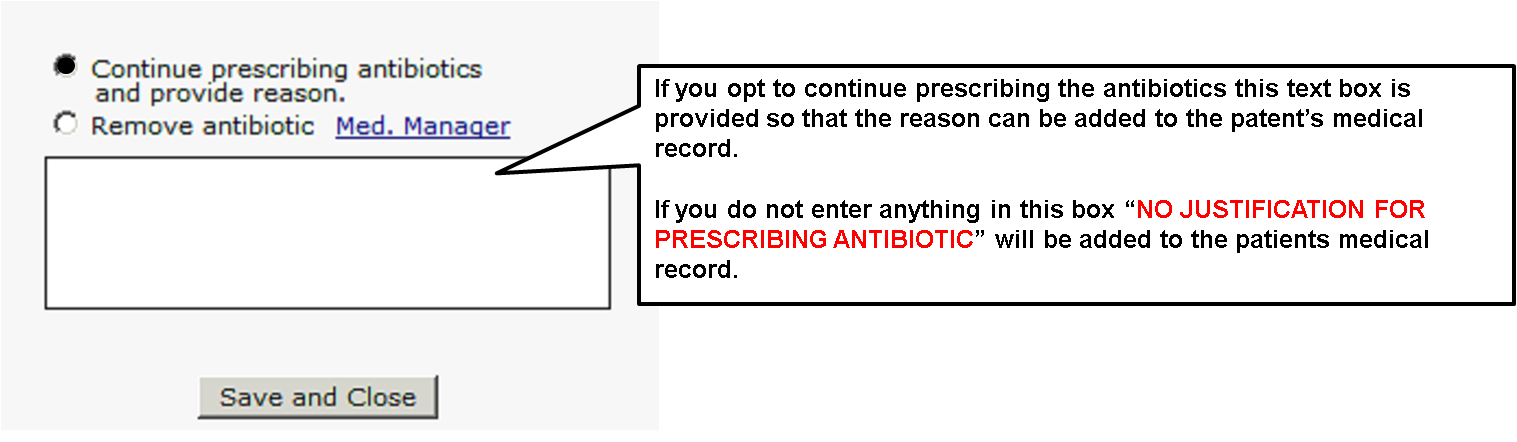
**

**Appendix E: Diagnosis code sets used in outcome assessments and clinical decision support**

**This is contained in an accompanying Microsoft Excel file.Appendix F: Example of Suggested Alternatives order set**

**Over-the-counter medications**

**Decongestants**

- Pseudoephedrine HCL (SUDAFED) 30 MG TABS

Two tablets every 6 hours as needed for nasal congestion. Dispense 50, Refills 0.

- Oxymetazoline HCl (AFRIN SINUS) 0.05% SOLN

One or two sprays in each nostril twice a day or as needed, but no more frequently than every 6 hours. Do not use for more than 3 days. Dispense 1 bottle, Refills 0.

**Antihistamines**

- Loratadine 10 MG TABS

One tablet by mouth once a day as needed. Dispense 30. Refills 0.

- DiphenhydrAMINE 25 MG TABS

Take one or two tablets by mouth every 4 to 6 hours as needed, not to exceed 6 doses in 24 hours. Dispense 24, Refills 0.

**Analgesics and antipyretics**

- Ibuprofen 200 MG TABS

One or two tablets by mouth every 6 hours as needed for aches and pains due to colds or sore throat or to reduce fever. Dispense 50, Refills 0.

- Acetaminophen 500 MG TAB

One or two tablets by mouth every 6 hours as needed for aches and pains due to colds or sore throat or to reduce fever. Do not take more than 8 pills (4000 MG) in one day. Dispense 50, Refills 0.

- Menthol (CEPACOL SORE THROAT) 3MG LOZG

Allow 1 lozenge to dissolve slowly in the mouth; may be repeated every 2 hours as needed for up to 2 days. Dispense 18, Refills 0.

**Cough suppressants and expectorants**

- Guaifenesin-DM 100-10 MG/5ML SYRUP

One or two teaspoons every 4 hours as needed for cough. Dispense 1 bottle, Refills 0.

- Guaifenesin 200 MG TABS

One or two tablets every 4 hours as needed for cough. Dispense 100, Refills 0.

**Prescription medications**

- Ipratropium Bromide (ATROVENT) 0.06% SOLN

Two sprays each nostril 4 times a day as needed for runny nose and sneezing for up to 4 days.

- Ibuprofen 600 MG TABS

One tablets by mouth every 6 hours as needed for aches and pains due to colds or sore throat or to reduce fever. Dispense 28, Refills 0.

- GUAIFENESIN-CODEINE (CHERATUSSIN AC) 100-10 MG/5ML SYRUP

One or two teaspoons every 4 hours as needed for cough. Dispense 180 ML, Refills 0.

- Benzonatate (TESSALON PERLES) 100 MG CAPS.

One capsule every 4 hours as needed for cough. Do not take more than 6 capsules in 1 day. Dispense 30. Refills 0.

- ALBUTEROL HFA 108 (90 BASE) MCG/ACT AERS

One or two inhalations every 6 hours as needed for cough. Dispense 1 inhaler. Refills 0.

**Patient information (will appear in patient instructions)**

☐ About Non-Specific Upper Respiratory Infection or “Common Cold”

**TEXT FOR PATIENT INSTRUCTIONS**

Non-Specific Upper Respiratory Infection or “Common Cold”

Your doctor has diagnosed you with a non-specific upper respiratory infection. This is also called the “common cold.” The symptoms of a cold include watery eyes, runny nose, nasal stuffiness, sneezing, scratchy or sore throat, fatigue, fever, muscle aches, and cough. Most colds last 1 to 2 weeks. Although you may feel bad, the common cold almost never causes serious illness.

Colds are caused by viruses. Cold viruses are spread through the air, through contact with people who have a cold, and on surfaces that have been touched by people with a cold. After you have caught a cold virus, it takes 2 or 3 days for you to develop symptoms. You can avoid getting and spreading colds by washing your hands frequently, avoiding other people with colds, avoiding touching your face, and coughing or sneezing into a tissue.

You cannot treat cold viruses directly, but you can treat the symptoms. Your doctor may have made specific recommendations for medications to help treat your symptoms.

In addition, you can soothe a sore throat by gargling with warm water. If you smoke, you should stop smoking and avoid smoke. You should avoid alcohol until your symptoms are gone.

**Antibiotics:** Antibiotics do not help colds. Antibiotics only kill bacteria, but they are not effective against viruses that cause colds. If you use unnecessary antibiotics, you run the risk of having diarrhea and yeast infections, having an allergic reaction,  and increasing your risk of having an infection later with antibiotic-resistant bacteria. Colored nasal discharge or sputum is a frequent symptom of the common cold and does not necessarily indicate a bacterial infection.

**You should contact your doctor if:**

- Your symptoms have not improved after 14 days
- You develop a high fever (above 102°F), confusion, difficulty breathing or swallowing, severe headache, pain in your face or forehead, severe fatigue, or a rash

**Appendix G: Sample Peer Comparison emails to providers**

**
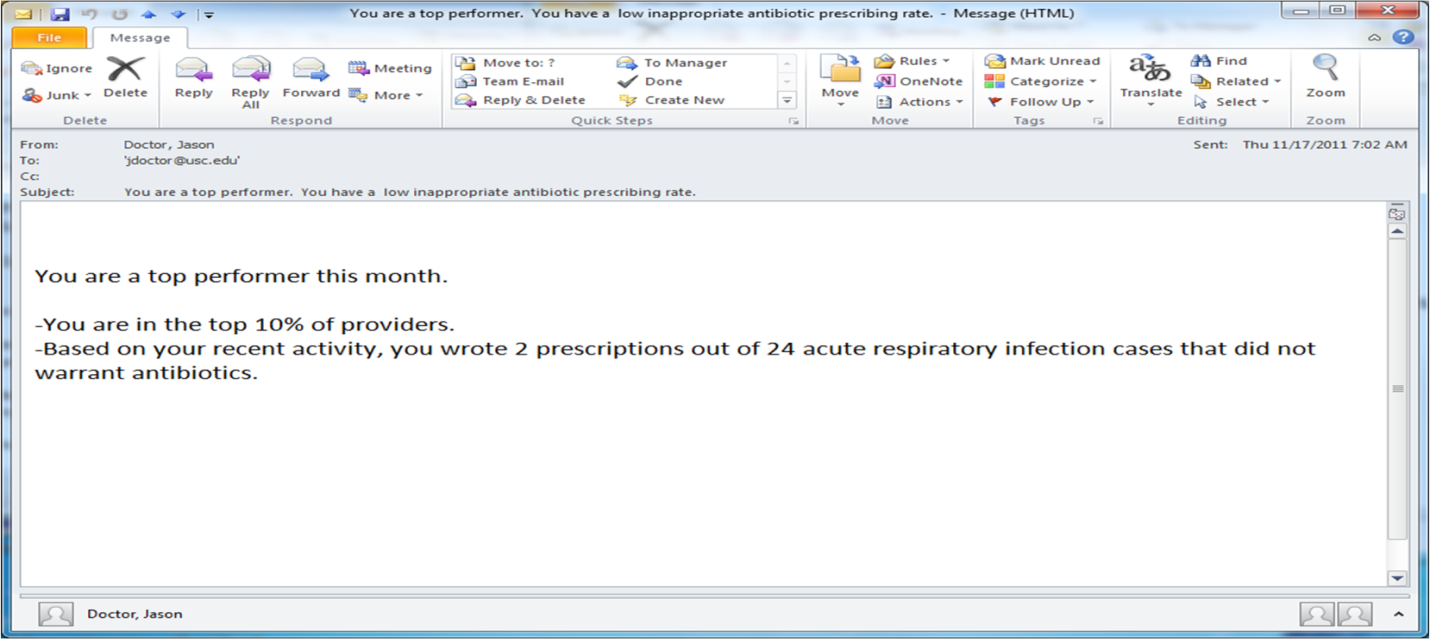
**

**
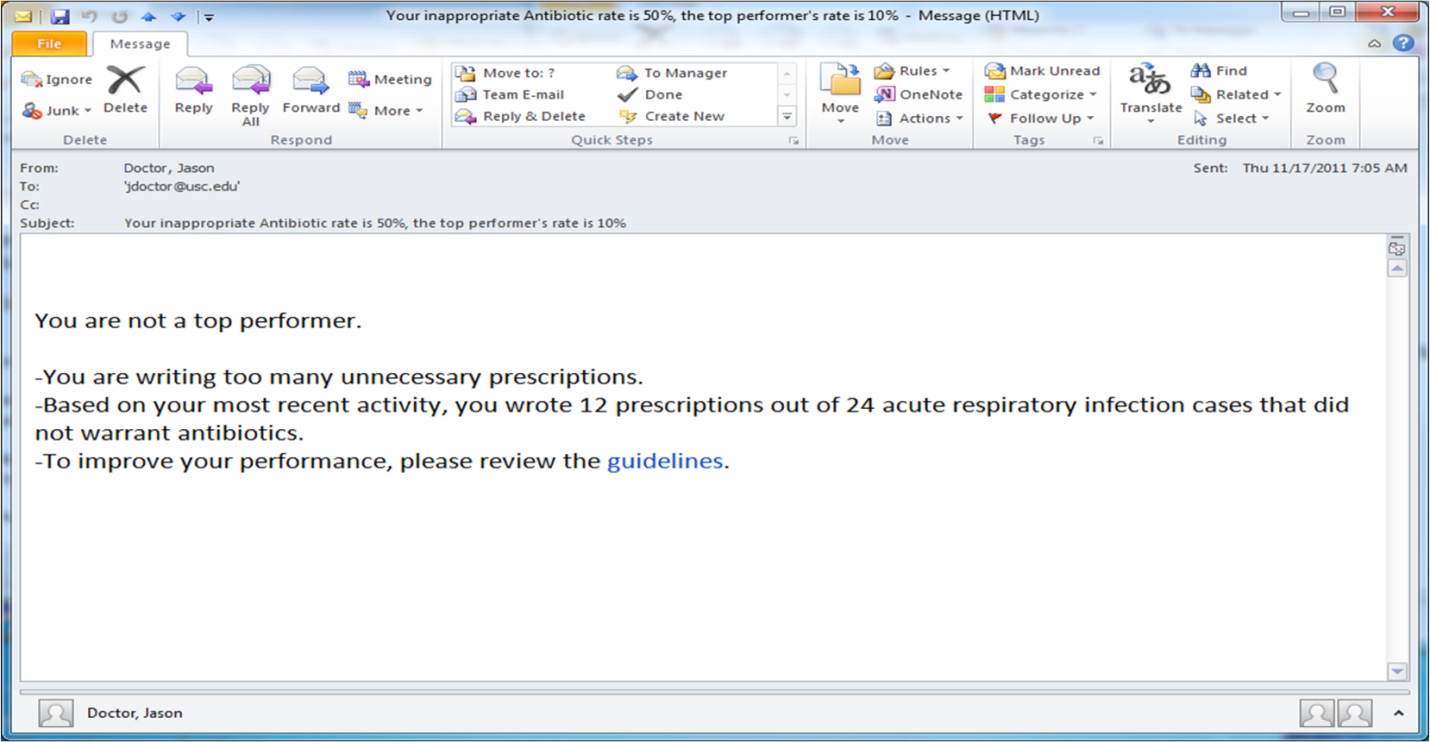
**

**Appendix H: Oral antibiotics included in outcome measurements**

| Cephalosporins | Other antimicrobials | |
| --- | --- | --- |
| Macrolides |  | Clindamycin |
| Penicillins |  | Linezolid |
| Quinolones |  | Telithromycin |
| Sulfonamides |  | Trimethoprim |
| Tetracyclines |  |  |
